# Parental Stress and Parental Ratings of Behavioral Problems of Enuretic Children

**DOI:** 10.3389/fneur.2019.01054

**Published:** 2019-10-04

**Authors:** Michele Roccella, Daniela Smirni, Pietro Smirni, Francesco Precenzano, Francesca Felicia Operto, Valentina Lanzara, Giuseppe Quatrosi, Marco Carotenuto

**Affiliations:** ^1^Department of Psychology, Educational Science and Human Movement, University of Palermo, Palermo, Italy; ^2^Department of Educational Sciences, University of Catania, Catania, Italy; ^3^Clinic of Child and Adolescent Neuropsychiatry, Department of Mental Health and Physical and Preventive Medicine, Università Degli Studi Della Campania “Luigi Vanvitelli”, Caserta, Italy; ^4^Child and Adolescent Neuropsychiatry, Medical School, University of Salerno, Fisciano, Italy; ^5^Dipartimento Promozione della Salute, Materno-Infantile, di Medicina Interna e Specialistica di Eccellenza “G. D'Alessandro”, Palermo, Italy

**Keywords:** primary monosymptomatic nocturnal enuresis (PMNE), parental stress, CBCL (child behavior checklist), parenting stress index-short form, self-rating scales

## Abstract

**Background:** Primary monosymptomatic nocturnal enuresis (PMNE) may have a stressful impact on the everyday life of children and parents, and it may represent a cumulative stress factor increasing feelings of “learned helplessness.”

**Methods:** The current study investigated parental stress in a group of parents (*n* = 330) of children affected by PMNE, compared to a group of parents (*n* = 330) of typical developing children (TDC). In addition, the study evaluated whether parents of PMNE children experience more emotional, social, and behavioral problems in their children, compared to parents of TDC. Finally, the study correlated frequency of enuresis with stress values and Child Behavior Checklist (CBCL) subscales and total stress with CBCL. Both groups were given The Parental Stress Inventory-Short Form (PSI-SF) and the Child Behavior Checklist (CBCL).

**Results:** Parents of PMNE children showed significantly higher stress level than parents of TDC. Nocturnal enuresis, as a demanding clinical condition difficult to control, represents a relevant stress factor. Mothers appeared as more vulnerable to stress than fathers. Parents of PMNE children reported higher behavioral and emotional problems, compared to reports of parents of TDC. PMNE children appeared to their parents as having lower competency in social activities, school performance, and social relationships than TDC. Moreover, they were rated as more withdrawn, anxious-depressed, more aggressive, inattentive, and with more somatic complaints than healthy children. It was always the mother who rated a significantly higher number of emotional, social, and behavioral problems compared to fathers. Correlational analysis showed that the higher the frequency of enuresis, the greater the parental stress level, the lower the social activities, school performance and relational competencies and the higher the emotional, social and behavioral problems in children, according to the parents' evaluations. The greater the parental stress level, the lower the competencies rated and the higher the behavioral problems detected by parents.

**Conclusion:** The physicians who deal with PMNE children have taken into account the stressful role and emotional dimensions of this clinical condition, both for children and mothers, in order to improve clinical management. Psychological support is needed for parents, and mothers especially, for a more functional stress management related to the PMNE.

## Background

Primary monosymptomatic nocturnal enuresis (PMNE), according to DSM-5 criteria (1), is a childhood clinical condition characterized by repeated voiding of urine into bed or clothes, whether involuntary or intentional, occurring during sleep (2), with many comorbid conditions such as visuomotor impairment (3), sleep disorders (4, 5), reading difficulties (6), primary headaches (7), and specific facial morphology (8). The behavior either (a) occurs at least twice a week for at least 3 consecutive months or (b) results in clinically significant distress or social, functional, or academic impairment (1). According to the ICD-10 Classification of Mental and Behavioral Disorders (9), a child is diagnosed with PMNE if there is at least one wetting event per month for children 7 years of age and older, and the bedwetting is not due to neurological disorders, abnormalities of the urinary tract, or epileptic attacks.

Such pathology may have a stressful impact on the everyday life of children and parents (10). The disappointments at each awakening after the enuretic episode, the repeated nocturnal controls, the impotence for a pathology that cannot be resolved despite the repeated medical consultations, the endless clinical and instrumental tests, the useless attempts to solve the problem, the embarrassment of every morning of all the protagonists, parents and children, and not least, the shame for a situation that could also be considered as a lack of commitment, everything can represent a continuous and relevant source of stress and “learned helplessness.”

In accordance with the learned helplessness model (11–13), any enuretic episode is characterized as an uncontrollable event that occurs outside the control of everyone, including the parents (14). Such an uncontrollable and unpredictable situation becomes a challenging and difficult condition to manage and an important cumulative stress factor that increases feelings of helplessness (15).

The comorbidity of monosymptomatic nocturnal enuresis with internalizing and externalizing problems, and attention deficit hyperactivity disorder in children has been studied (16–22). However, parental reports of the problem behavior of enuretic children and the stress of mothers and fathers have hardly been considered. Moreover, most research has been derived primarily from maternal report measures, while paternal views were relatively neglected.

Therefore, the first goal of the current study was to investigate parental stress in a group of mothers and fathers of children with PMNE, compared to a group of mothers and fathers of TDC. The second goal was to examine the presence of problematic behaviors in the ratings of the parents of enuretic children with respect to the reports of TDC's parents. The third goal was to study the correlation between the frequency of enuresis episodes, parental stress and the parents' evaluations of their children's problem behaviors.

The hypotheses were: (1) the parents of enuretic children are more stressed than the parents of TDC; (2) the parents of enuretic children rate more problematic behaviors in their children than the parents of TDC; (3) monthly frequency of enuresis, stress and parental ratings of behavioral problems are correlated variables.

## Materials and Methods

### Study Population

The study population included two groups of parental couples. The first group consisted of 165 sets of parents to children−89 boys and 76 girls—aged between 5.8 and 11.6 years, who had displayed monthly frequency of PMNE during the last 4 weeks of their time as inpatients from May 2015 until November 2017 at the Clinic for Child and Adolescent Neuropsychiatry of the University of Campania “Luigi Vanvitelli” and at the Clinic of Child and Adolescent Neuropsychiatry at University of Palermo. PMNE diagnosis was made by trained child and adolescent neuropsychiatrists (MC, MR), according to the international criteria (2).

The second group consisted of 165 sets of parents of TDC, 84 boys and 81 girls, aged between 5.5 and 11.4 years, recruited in several public schools, from the same geographical area, as part of a study on the typical neurological development of primary school children.

In both groups, the age of the parents was ranged between 34 and 43 years (mean age 37.2 ± 7.5). Participants were all Caucasian, Italian speakers, and they came from the same middle socioeconomic status.

### Parenting Stress Index-Short Form (PSI-SF)

To assess parents' stress, the Italian version of the PSI-SF was used (23–27). The PSI-SF is a self-rating standardized tool that yields scores for parental stress and three different domains: (1) Parental Distress; (2) Parent-Child Interaction; (3) Difficult Child.

The Parental Distress subscale assesses the parent's stress arising from the parental role as such (e.g. “I feel trapped by my responsibilities as a parent”). The Parent-Child interaction subscale evaluates the stress that derives specifically from the interaction with the child (e.g., “my child smiles at me much less than I expected”). The Difficult Child subscale measures the stress that comes from managing a child that appears more problematic than the parent expected (e.g., “my child turned out to be a bigger problem than I expected”).

PSI-SF has 36 items based on a five-point Likert scale where each value corresponds to a specific statement (1 = completely disagree; 5 = completely agree). The subscale scores range from 12 to 60, and the Total Stress score ranges from 36 to 180. The higher the score, the greater the level of parental stress.

The PSI-SF showed high internal consistency (Cronbach's alpha 0.92), and its validity has been reported in parents of children with chronic medical conditions. The PSI-SF has been widely used, and psychometric evidence supports its reliability and validity. In the current study, the three subscales have adequate internal reliability as measured by Cronbach's alpha ranging from 0.57 to 0.70.

### Child Behavior Checklist (CBCL)

A self-rating scale was administered to evaluate emotional, social, and behavioral problems in children, according to the parents' point of view and evaluation. The CBCL was used as a standardized instrument filled out by parents about their child's behavior during the past 6 months, scoring on a three-step response scale (0–2) (28, 29).

The first part of the scale examines children's social competencies, their participation in sports, home and school activities, games, and relationships with peers, siblings, and parents (activities, school, competence). The second part is focused on eight problematic behavioral and emotional areas, including withdrawal, somatic complaints, anxious/depressive, social problems, thought problems, attention problems, delinquent behavior, and aggressive behavior. Withdrawal, somatic complaints and anxious/depressive scales can be grouped into the internalizing factor. Delinquent behavior and aggressive behavior can be grouped into the externalizing factor. Social, attention, and thought problems are included in the total problems scale. All items together form the total score scale. The higher the score, the more relevant the behavior problem rated.

### Ethical Approval Study Design

All participants gave their written informed consent. The study was conducted according to the Ethical Principles of Helsinki Declaration for experiments involving humans (30). The Departmental Ethic Committee of the University of Campania “Luigi Vanvitelli” approved the study (Prot. n. 13891; EudraCT number 2015-001159-66).

### Statistical Analysis

The *t*-test mean comparison was carried out to compare the performance in PSI-SF and CBCL of parents of children with enuresis problems, and TDC parents. Further analyses were performed to check if there were differences between mother's and father's scores among parents of children with enuresis disorders. Furthermore, an analysis of parents of daughters and sons has been carried out, to investigate whether gender can affect stress level and different scores in the CBCL. Additional correlational analyses were performed to verify if there was a relationship between the frequency of enuresis episodes and the level of parental stress, and whether the frequency of enuresis was related to the CBCL subscales. Further correlational analyses investigated the relation between total stress score and CBCL performances. For all analyses, *p* ≤ 0.05 were considered as statistically significant.

## Results

Table 1 shows that there were no significant differences between mothers and fathers of all participants in this study in the stress measurement scale and its sub-scales. As for the Child Behavior Checklist (CBCL), significant differences emerged only on some indexes (Activities, Competence, Anxious/depressed, Thought, Attention, Aggressive) and with an effect size between small and medium. In all the sub-scales where differences between mothers and fathers were found, mothers always had a higher score than fathers. The higher difference between mothers and fathers was shown on Anxious/depressed scale [Anxious/depressed scale: mean of Mothers 63.03 ± 11.18 vs. mean Fathers 58.68 ± 8.61, *t*_(328)_ = 3.96, *p* < 0.001, *d* = 0.44] (Table 1).

**Table 1:** Mean comparison between mothers and fathers in the Parent Stress Index-Short Form (PSI-SF) and Child Behavior Checklist (CBCL) scales.

	**Mothers**		**Fathers**		***t***	***p***	**Cohen's d**
**Scale**	***M***	***SD***	***M***	***SD***	**(*df* = 328)**		
**PSI-SF**							
Stress total score	70.94	23.81	68.73	22.63	0.86	0.391	0.09
Parental distress	22.36	7.68	22.54	8.28	0.21	0.831	0.02
Parent-child interaction	24.29	9.24	22.93	8.51	1.39	0.164	0.15
Difficult child	24.28	8.47	23.25	7.43	1.17	0.242	0.13
**CBCL**							
Activities	34.46	6.91	31.87	6.72	3.45	<0.001*	0.38
School	44.84	6.60	45.76	6.32	1.29	0.197	0.14
Competence	36.55	6.85	34.83	6.79	2.29	0.023*	0.25
Withdrawn	59.73	9.23	58.27	7.99	1.54	0.125	0.17
Somatic complaints	60.53	10.20	61.25	9.54	0.66	0.508	0.07
Anxious/Depressed	63.03	11.18	58.68	8.61	3.96	<0.001*	0.44
Social	59.29	9.10	59.28	10.03	0.01	0.992	0.00
Thought	56.35	7.89	54.34	6.57	2.51	0.012*	0.28
Attention	61.25	9.72	58.38	8.90	2.80	0.005*	0.31
Delinquent	55.95	6.50	55.04	6.05	1.32	0.189	0.14
Aggressive	57.81	8.45	55.74	7.09	2.41	0.016*	0.26
Internalizing	61.42	13.48	58.68	12.06	1.95	0.052	0.21
Externalizing	54.79	11.09	53.35	9.12	1.29	0.199	0.14
Total score	59.64	13.73	57.38	11.65	1.61	0.108	0.18

*PSI-SF, Parent Stress Index-Short Form; CBCL, Child Behavior Checklist; t, t-test; p, p-value; df, degrees of freedom; M, mean; SD, standard deviation*.

* *p < 0.05 statistically significant*.

As expected, comparing the performance of parents of children with enuresis and parents of TDC, means of all the parameters measured by the stress scale and by the CBCL were statistically significant and with an effect sizes between large and very large (Table 2).

**Table 2:** Mean comparison between parents of TDC and parents of children with enuresis in the Parent Stress Index-Short Form (PSI-SF) and Child Behavior Checklist (CBCL) scales.

	**TDC parents**		**Enuresis parents**		***t***	***p***	**Cohen's d**
**Scale**	***M***	***SD***	***M***	***SD***	**(*df* = 658)**		
**PSI-SF**							
Stress total score	48.09	4.88	91.58	10.57	67.86	<0.001*	5.28
Parental distress	15.87	2.02	29.04	6.07	37.40	<0.001*	2.91
Parent-child interaction	15.49	2.21	31.72	4.72	56.57	<0.001*	4.40
Difficult child	16.72	1.66	30.81	5.07	47.98	<0.001*	3.73
**CBCL**							
Activities	35.32	6.22	31.01	6.95	8.39	<0.001*	0.65
School	48.23	4.93	42.44	6.53	12.85	<0.001*	1.00
Competence	38.49	6.02	32.90	6.53	11.43	<0.001*	0.89
Withdrawn	53.33	5.36	64.60	7.60	22.01	<0.001*	1.71
Somatic complaints	53.92	6.23	67.76	7.79	25.20	<0.001*	2.12
Anxious/Depressed	53.59	4.93	67.92	8.98	25.41	<0.001*	1.98
Social	53.63	5.88	64.82	9.27	18.52	<0.001*	1.44
Thought	51.31	3.44	59.26	7.96	16.65	<0.001*	1.29
Attention	53.29	5.28	66.19	8.12	24.19	<0.001*	1.88
Delinquent	51.97	3.63	58.93	6.45	17.08	<0.001*	1.33
Aggressive	51.78	3.21	61.64	8.01	20.76	<0.001*	1.62
Internalizing	50.22	8.80	69.71	7.96	29.84	<0.001*	2.32
Externalizing	47.35	7.22	60.63	7.96	22.45	<0.001*	1.75
Total score	48.23	8.35	68.54	7.11	33.64	<0.001*	2.62

*PSI-SF, Parent Stress Index-Short Form; CBCL, Child Behavior Checklist; t, t-test; p, p-value; df, degrees of freedom; M, mean; SD, standard deviation*.

* *p < 0.05 statistically significant*.

Further analysis was performed to identify differences between mothers and fathers in relation to the sex of the child. Interestingly, it emerged that, among parents of daughters with enuresis disorders, mothers had significantly higher average scores on the stress scale compared to fathers. In particular, significant differences in the stress total score in the parent-child interaction and in the difficult child scales have been highlighted [*Stress total score:* mean of Mothers 93.41 ± 11.08 vs. mean of Fathers 89.74 ± 9.38, *t*_(312)_ = 3.17, *p* = 0.002, *d* = 0.36; *parent-child interaction:* mean of Mothers 32.32 ± 4.35 vs. mean of Fathers 30.46 ± 4.38, *t*_(312)_ = 3.77, *p* = 0.001, *d* = 0.43; *difficult child:* mean of Mothers 32 ± 5.67 vs. mean of Fathers 30.02 ± 4.52, *t*_(312)_ = 3.42, *p* < 0.001, *d* = 0.39]. However, no significant difference has emerged in the parental distress scale. Furthermore, mothers of daughters reported significant higher scores than fathers in several subscales of the CBCL: Activities, Competence, Withdrawn, Anxious/depressed, Thought, Attention, Aggressive, Internalizing, Externalizing, Total score [*Activities:* mean of Mothers 33 ± 6.36 vs. mean of Fathers 29.26 ± 6.58, *t*_(312)_ = 5.12, *p* < 0.001, *d* = 0.58; *Competence:* mean of Mothers 34 ± 5.97 vs. mean of Fathers 31.89 ± 6.37, *t*_(312)_ = 3.87, *p* < 0.001, *d* = 0.44; *Withdrawn:* mean of Mothers 65.85 ± 7.27 vs. mean of Fathers 62.33 ± 7.67, *t*_(312)_ = 4.17, *p* < 0.001, *d* = 0.47; *Anxious/depressed:* mean of Mothers 71.08 ± 7.85 vs. mean of Fathers 63.23 ± 8.87, *t*_(312)_ = 8.30, *p* < 0.001, *d* = 0.94; *Thought:* mean of Mothers 61.60 ± 7.18 vs. mean of Fathers 57.29 ± 7.85, *t*_(312)_ = 5.08, *p* < 0.001, *d* = 0.57; *Attention:* mean of Mothers 67.92 ± 5.82 vs. mean of Fathers 63.33 ± 9.17, *t*_(312)_ = 5.29, *p* < 0.001, *d* = 0.60; *Aggressive:* mean of Mothers 63.28 ± 7.17 vs. mean of Fathers 59.93 ± 8.39, *t*_(312)_ = 3.80, *p* < 0.001, *d* = 0.43; *Internalizing*: mean of Mothers 72.01 ± 4.63 vs. mean of Fathers 66.12 ± 10.36, *t*_(312)_ = 6.50, *p* < 0.001, *d* = 0.73; *Externalizing*: mean of Mothers 62.60 ± 6.19 vs. mean of Fathers 58.13 ± 9.76, *t*_(312)_ = 4.85, *p* < 0.001, *d* = 0.55; *Total score*: mean of Mothers 70.68 ± 2.94 vs. mean of Fathers 65.20 ± 9.71, *t*_(312)_ = 6.77, *p* < *0.0*01, *d* = 0.76].

As for the parents of sons with enuresis disorders, mothers had significantly higher mean scores on the stress scale compared to fathers. Particularly, significant differences in the stress total score in the parent-child interaction and in the difficult child scales were highlighted [*Stress total score:* mean of Mothers 93.04 ± 10.045 vs. mean of Fathers 90.12 ± 10.72, *t*_(344)_ = 2.56, *p* = 0.011, *d* = 0.28; *parent-child interaction:* mean of Mothers 33.22 ± 4.65 vs. mean of Fathers 30.80 ± 4.87, *t*_(344)_ = 4.72, *p* = 0.001, *d* = 0.51; *difficult child:* mean of Mothers 31.45 ± 5.42 vs. mean of Fathers 29.83 ± 4.22, *t*_(344)_ = 3.10, *p* = 0.002, *d* = 0.33]. However, no significant difference has emerged in the parental distress scale. Furthermore, mothers of sons reported significantly higher scores than fathers in several subscales of the CBCL: Activities, Competence, Withdrawn, Anxious/depressed, Thought, Attention, Delinquent, Aggressive, Internalizing, Externalizing, Total score [*Activities:* mean of Mothers 32.95 ± 7.27 vs. mean of Fathers 28.89 ± 6.36, *t*_(344)_ = 5.53, *p* < 0.001, *d* = 0.59; *Competence:* mean of Mothers 33.95 ± 6.51 vs. mean of Fathers 31.27 ± 6.59, *t*_(344)_ = 3.80, *p* < 0.001, *d* = 0.41; *Withdrawn:* mean of Mothers 65.91 ± 7.97 vs. mean of Fathers 64.14 ± 6.89, *t*_(344)_ = 2.21, *p* = 0.028, *d* = 0.24; *Anxious/depressed:* mean of Mothers 72.61 ± 7.05 vs. mean of Fathers 64.54 ± 8.31, *t*_(344)_ = 9.74, *p* < 0.001, *d* = 1.05; *Thought*: mean of Mothers 60.37 ± 8.62 vs. mean of Fathers 57.84 ± 7.24, *t*_(344)_ = 2.96, *p* = 0.003, *d* = 0.32; *Attention:* mean of Mothers 69.85 ± 5.67 vs. mean of Fathers 63.49 ± 9.03, *t*_(344)_ = 7.84, *p* < 0.001, *d* = 0.84; *Delinquent*: mean of Mothers 60.02 ± 6.26 vs. mean of Fathers 58.57 ± 6.65, *t*_(344)_ = 2.09, *p* = 0.037, *d* = 0.22; *Aggressive:* mean of Mothers 63.84 ± 8.11 vs. mean of Fathers 59.49 ± 7.33, *t*_(344)_ = 5.23, *p* < 0.001, *d* = 0.56; *Internalizing:* mean of Mothers 72.79 ± 4.59 vs. mean of Fathers 67.75 ± 8.67, *t*_(344)_ = 6.76, *p* < 0.001, *d* = 0.73; *Externalizing:* mean of Mothers 63.13 ± 6.77 vs. mean of Fathers 58.59 ± 8.19, *t*_(344)_ = 5.62, *p* < 0.001, *d* = 0.60; *Total Score:* mean of Mothers 71.52 ± 2.60 vs. mean of Fathers 66.61 ± 8.17, *t*_(344)_ = 7.53, *p* < 0.001, *d* = 0.81]. Interestingly, fathers of sons in this case reported a significantly higher score than mothers in the CBCL subscale of School [*School:* mean of Mothers 41.34 ± 6.57 vs. mean Fathers 43.61 ± 7.08, *t*_(344)_ = 3.09, *p* = 0.002, *d* = 0.33].

Furthermore, the correlational analyses revealed that the monthly frequency of enuresis episodes correlates with all the dimensions of stress. All the correlations are highly significant and positive (Table 3). With regard to the analysis of the correlation between frequency of enuresis and CBCL subscales, it emerged that all the subscales were highly significantly correlated. Three were negatively correlated (i.e., Activities, School, Competence) and all others were positively correlated to the frequency of episodes of enuresis. However, no correlation emerged between episodes of enuresis and the age of the child (*r* = 0.04; *p* = 0.28) (Table 3).

**Table 3:** Correlation between enuresis monthly frequency, total stress scores and child age, indexes from Parent Stress Index-Short Form (PSI-SF), and scores of Child Behavior Checklist (CBCL) scales.

	**Enuresis monthly frequency**		**Total stress score**	
	***r***	***p***	***r***	***p***
Child age	0.04	0.28	0.02	0.69
**PSI-SF**				
Stress total score	0.82	<0.001*	−	−
Parental distress	0.70	<0.001*	−	−
Parent-child interaction	0.81	<0.001*	−	−
Difficult child	0.78	<0.001*	−	−
**CBCL**				
Activities	−0.25	<0.001*	−0.28	<0.001*
School	−0.42	<0.001*	−0.43	<0.001*
Competence	−0.35	<0.001*	−0.37	<0.001*
Withdrawn	0.57	<0.001*	0.61	<0.001*
Somatic complaints	0.62	<0.001*	0.64	<0.001*
Anxious/Depressed	0.61	<0.001*	0.68	<0.001*
Social	0.51	<0.001*	0.54	<0.001*
Thought	0.47	<0.001*	0.52	<0.001*
Attention	0.60	<0.001*	0.66	<0.001*
Delinquent	0.49	<0.001*	0.53	<0.001*
Aggressive	0.56	<0.001*	0.61	<0.001*
Internalizing	0.66	<0.001*	0.72	<0.001*
Externalizing	0.58	<0.001*	0.63	<0.001*
Total score	0.70	<0.001*	0.75	<0.001*

*PSI-SF, Parent Stress Index-Short Form; CBCL, Child Behavior Checklist; r, correlation's coefficient; p, p-value; df, degrees of freedom*.

* *p < 0.05 statistically significant*.

A further correlational analysis between total stress score and CBCL scale indices revealed the presence of a highly significant correlation. Activities, School, and Competence subscales were negatively correlated, and all the others were positively correlated to the total stress score. No correlation emerged between total stress score and the age of the child (*r* = 0.02; *p* = 0.69) (Table 3).

## Discussion

The current study investigated parental stress in a group of mothers and fathers of children affected by PMNE, compared to a group of parents of TDC. In addition, the study evaluated whether parents of enuretic children experience more emotional, social and behavioral problems in their children, compared to TDC parents. Finally, the study correlated monthly frequency of enuresis with stress values and CBCL subscales, and total stress with CBCL.

As in our first hypothesis, parents of enuretic children showed a significantly higher stress level than parents of TDC, both in total stress and in individual PSI-SF subscales. Their stress scores doubled those of parents of healthy children. However, the most marked differences were in the Parent-Child Interaction and in the Difficult Child subscales, while the Parental Distress subscale showed the lowest differences. What is perceived as a more relevant source of stress is being the parents of a difficult child, namely of an enuretic child, rather than being a parent as such.

These findings confirmed our hypothesis, according to which primary nocturnal enuresis represents a demanding clinical condition, difficult to control, and significantly impacting on family life style. Probably, every new enuretic crisis, as an uncontrollable event, becomes for parents a new stressful event that is added to the previous ones with a cumulative effect (15).

Data are consistent with studies relating primary nocturnal enuresis to parenting stress (10, 31, 32) and showing more reported problem behaviors in parents of children with enuresis (10), and a negative impact on quality life of mothers (31), on school performance (33), on attention and hyperactivity (16), and on internalizing and externalizing behavior problems (17–20). More generally, our data were in line with a large body of literature showing that parenting a child with developmental problems or a chronic pathological condition can be a significant source of parental stress (25, 34–42).

Comparing the level of stress within groups of parents of enuretic children, mothers showed significantly higher values than fathers in both total stress scores and parent-child interaction and difficult child subscales. Mothers appeared as the more stressed parent both when the enuretic child was a girl or a boy, showing a higher level of stress in all domains except for Parental Stress.

In sum, parents of enuretic children showed a higher stress index than the parents of TDC. Mothers were more vulnerable to stress than fathers, and once again, the source of stress does not seem the parental role as such but rather the relationship with a problem child.

Interestingly, comparing the entire population of mothers (i.e., PMNE and TDC mothers) to the entire population of fathers (i.e., PMNE and TDC fathers) on the stress level there were no significant differences either in total stress or in each stress subscale of PSI-SF. This result further supports the evidence that it is not being a mother that creates stress, but being the mother of a difficult enuretic child. Moreover, episodes of enuresis persisting for too long a time can favor dysfunctional, psychopathological, or depressive reactions. Especially considering that mothers are generally the main protagonists of their children's care, as primary caregiver.

Several studies have shown that mothers can usually feel more the burden of caring for their children than fathers (42, 43), especially in the care of disabled or problematic children (44, 45). Mothers of autistic children have been found to have higher stress levels than fathers, as they are more often the primary caregiver (44, 46). While mothers face greater difficulties due to physical problems related to the care of disabled children, fathers are more stressed by other conditions, such as career problems or financial difficulties (47, 48). After each enuresis episode, the mothers take care of the cleaning, the laundry, and the prevention of skin diseases, as well as the control of the child's reactions, and other members of the family.

In clinical practice, this suggests that the mother's experience and feelings cannot be neglected in an enuresis treatment program.

Examining the responses at CBCL, parents of PMNE children reported higher behavioral and emotional problems, compared to reports of parents of TDC. Enuretic children appeared to their parents as having lower competency in social activities, school performance, and social relationships. Moreover, they were perceived as more withdrawn, anxious-depressed, more aggressive, inattentive, and with more somatic complaints than healthy children. The differences also concerned social problems and thought (“Can't get his/her mind off certain thoughts/ obsessions” and “Repeats acts over and over”). Both externalizing and internalizing scales were higher in the enuretic children parents' reports, even if the internalizing scale reported significantly higher scores. These findings are consistent with our second hypothesis according to which the parents of enuretic children rate more problematic behaviors in their children than the parents of TDC.

It can be hypothesized that the reoccurrence of enuresis episodes fosters in the parents a sort of negative attitude that tends to generalize on a wider repertoire of child's behaviors. Enuretic child in turn could also develop a low self-esteem that favors a wide range of dysfunctional behaviors of social withdrawal, emotional lability, attention instability, and poor social and relational expansion. It is even more likely that the parents' “generalizing” attitude and children's behavior mutually reinforce each other according to a circular logic.

Interestingly, it is always the mother who rated a significantly higher number of emotional, social, and behavioral problems using a more negative perspective on child behavior compared to fathers. Therefore, their perception of children's behavior, regardless of gender, is more negative compared to the perception of fathers and parents with TDC (both fathers and mothers). These data appeared consistent with studies showing that mothers report more problems relating to externalizing behaviors and attention deficit hyperactivity disorder symptoms than controls (16, 19, 20).

This further suggests the need to think about specific training and psychological support programs for parents, especially for the mother, to re-elaborate the enuretic child's problem in more realistic terms. An unrealistic or exaggeratedly negative mother's perception of the child's behaviors can reinforce a low self-esteem in the child, creating a vicious circle in which the child's behavior and the mother's attitude negatively reinforce each other.

The correlational analyses showed a significant correlation between the stress values on PSI-SF and monthly frequency of enuresis episodes. Both total stress and all the domains of PSI-SF positively correlated with monthly frequency of enuretic episodes. The higher the frequency of enuresis, the greater the stress level.

Furthermore, the monthly frequency of enuresis and all the CBCL subscales evaluated by the parents were significantly correlated: the competence scales negatively, all the other scales positively. The more the number of enuretic episodes increases, the more the parents express “negative” judgments toward their children. It seems as if the emotional reactions to the enuretic facts tended to be generalized to other behaviors and acted as a deforming prism detecting above all the negative behaviors.

There was not a correlation between age of the child and monthly episodes of enuresis frequency.

Correlational analysis, therefore, confirmed our third hypothesis according to which frequency of enuresis correlated with parental stress values and CBCL subscales and total parental stress correlated with CBCL.

Two practical implications may be considered. First, in the treatment programs of enuretic children, due account must be taken of the emotional aspects of such pathology that can become the source of stress both for parents and for children themselves. It is necessary to provide specific psychological interventions for stress management, especially considering that the stress of parents can affect the psychosocial development of children in developmental age (49).

Second, a theoretical framework is required to support psychological programs and training for parents, and mothers especially, for a more functional stress coping.

In addition, several recent studies have also focused on investigating the therapeutic aspects of the enuresis. For example, Ferrara et al. have recently investigated the impact of a motivational therapy on the outcomes for individuals diagnosed with nocturnal enuresis (50). The authors showed that motivational therapy could help positive effects on compliance to treatment and adherence to pharmacological therapy. Furthermore, a recent review investigated studies on efficacy of enuresis alarm and desmopressin therapy in managing pediatric monosymptomatic enuresis (51). The results revealed that alarm therapy and desmopressin were comparable in efficacy by substantially reducing wet nights in enuretic children (47).

The following limitation should be considered when interpreting the results. The study examines emotional, social, and behavioral problems in children with PMNE, compared to TDC, according to the parents' point of view.

However, we do not know to what extent behavioral problems reported by parents, by a self-rating scale, are real problems of children, or rather mainly express the perception that parents have of their children's behavior. Further research could investigate the extent to which parents' evaluations show real behavioral problems of children, or result mainly from a negative generalized attitude toward the general behavior of the child and their difficulty in managing an enuretic child.

Moreover, further research is needed for a broader understanding of the relationship of parental evaluation, in particular of mothers, and the emotional and behavioral problems of the enuretic child.

## Data Availability Statement

The datasets generated for this study are available on request to the corresponding author.

## Ethics Statement

The studies involving human participants were reviewed and approved by The Departmental Ethic Committee of the Università degli Studi della Campania Luigi Vanvitelli approved the study (Prot. n. 13891; EudraCT No. 2015-001159-66). The patients/participants provided their written informed consent to participate in this study. The work has been carried out in accordance with The Code of Ethics of the World Medical Association (https://www.wma.net/policies-post/wmadeclaration-of-helsinki-ethicalprinciples-for-medical-researchinvolving-human-subjects/) (Declaration of Helsinki) for experiments involving humans.

## Author Contributions

MR, MC, DS, PS, and FO: substantial contributions to the design of the work. GQ, VL, and FP: data collection. DS and PS: data analysis and data interpretation. FO, DS, MC, MR, and PS: drafting the paper. MR, DS, PS, FP, FO, VL, GQ, and MC: revising the paper. All the authors read and approved the final manuscript.

### Conflict of Interest

The authors declare that the research was conducted in the absence of any commercial or financial relationships that could be construed as a potential conflict of interest.

## References

[1] American Psychiatric Association The Diagnostic and Statistical Manual of Mental Disorders. 5th ed. Washington, DC: American Psychiatric Association (2013).

[2] Nevéus T von Gontard A Hoebeke P Hjälmås K Bauer S Bower W. The standardization of terminology of lower urinary tract function in children and adolescents: report from the Standardisation Committee of the International Children's Continence Society. J Urol. (2006) 176:314–24. 10.1016/S0022-5347(06)00305-3 16753432

[3] Esposito M Gallai B Parisi L Roccella M Marotta R Lavano SM. Visuomotor competencies and primary monosymptomatic nocturnal enuresis in prepubertal aged children. Neuropsychiatr Dis Treat. (2013) 9:921–6. 10.2147/NDT.S46772 23847418 PMC3700782

[4] Esposito M Gallai B Parisi L Roccella M Marotta R Lavano SM. Primary nocturnal enuresis as a risk factor for sleep disorders: an observational questionnaire-based multicenter study. Neuropsychiatr Dis Treat. (2013) 9:437–43. 10.2147/NDT.S43673 23579788 PMC3621720

[5] Roccella M Marotta R Operto FF Smirni D Precenzano F Bitetti I. NREM sleep instability in pediatric migraine without aura. Front Neurol. (2019) 10:932. 10.3389/fneur.2019.00932 31551903 PMC6736572

[6] Esposito M Carotenuto M Roccella M. Primary nocturnal enuresis and learning disability. Minerva Pediatr. (2011) 63:99–104. 21487372

[7] Carotenuto M Esposito M Pascotto A. Migraine and enuresis in children: an unusual correlation? Med Hypotheses. (2010) 75:120–2. 10.1016/j.mehy.2010.02.004 20185246

[8] Carotenuto M Esposito M Pascotto A. Facial patterns and primary nocturnal enuresis in children. Sleep Breath. (2011) 15:221–7. 10.1007/s11325-010-0388-6 20607423

[9] Organization WH The ICD-10 Classification of Mental and Behavioural Disorders: Clinical Descriptions and Diagnostic Guidelines. Geneva: World Health Organization (1992).

[10] De Bruyne E Van Hoecke E Van Gompel K Verbeken S Baeyens D Hoebeke P. Problem behavior, parental stress and enuresis. J Urol. (2009) 182:2015–21. 10.1016/j.juro.2009.05.102 19695644

[11] Lazarus RS Folkman S Stress, Appraisal, and Coping. New York, NY: Springer publishing company (1984).

[12] Seligman MEP Helplessness: On Depression, Development, and Death. San Francisco, CA: WH Freeman (1975).

[13] Abramson LY Seligman MEP Modeling psychopathology in the laboratory: history and rationale. In: Psychopathology: Experimental Models. San Francisco, CA: WH Freeman (2001). p. 1–26.

[14] Technow JR Hazel NA Abela JRZ Hankin BL. Stress sensitivity interacts with depression history to predict depressive symptoms among youth: prospective changes following first depression onset. J Abnorm Child Psychol. (2015) 43:489–501. 10.1007/s10802-014-9922-5 25123081 PMC4423556

[15] Nederhof E Schmidt M V Mismatch or cumulative stress: toward an integrated hypothesis of programming effects. Physiol Behav. (2012) 106:691–700. 10.1016/j.physbeh.2011.12.008 22210393

[16] Baeyens D Roeyers H Hoebeke P Verte S Van Hoecke E Vande WJ. Attention deficit/hyperactivity disorder in children with nocturnal enuresis. J Urol. (2004) 171:2576–9. 10.1097/01.ju.0000108665.22072.b2 15118422

[17] Friman PC Handwerk ML Swearer SM McGinnis JC Warzak WJ. Do children with primary nocturnal enuresis have clinically significant behavior problems? Arch Pediatr Adolesc Med. (1998) 152:537–9. 10.1001/archpedi.152.6.537 9641705

[18] Kodman-Jones C Hawkins L Schulman SL. Behavioral characteristics of children with daytime wetting. J Urol. (2001) 166:2392–5. 10.1016/S0022-5347(05)65599-1 11696795

[19] Van Hoecke E De Fruyt F De Clercq B Hoebeke P Vande Walle J. Internalizing and externalizing problem behavior in children with nocturnal and diurnal enuresis: a five-factor model perspective. J Pediatr Psychol. (2005) 31:460–8. 10.1093/jpepsy/jsj037 15905418

[20] Van Hoecke E Hoebeke P Braet C Walle J Vande. An assessment of internalizing problems in children with enuresis. J Urol. (2004) 171:2580–3. 10.1097/01.ju.0000110521.20103.14 15118423

[21] Tsai J-D Wang I-C Chen H-J Sheu J-N Li T-C Tsai HJ. Trend of nocturnal enuresis in children with attention deficit/hyperactivity disorder: a nationwide population-based study in Taiwan. J Investig Med. (2017) 65:370–5. 10.1136/jim-2016-000223 27733442

[22] Ohtomo Y. Atomoxetine ameliorates nocturnal enuresis with subclinical attention-deficit/hyperactivity disorder. Pediatr Int. (2017) 59:181–4. 10.1111/ped.13111 27501068

[23] Abidin RR Parenting Stress Index-Short Form Manual. Los Angeles, CA: Western Psychological Services (1990). 10.1037/t02445-000

[24] Abidin RR Parenting Stress Index Third Edition: Professional Manual. Lutz, FL: Psychological Assessment Resources (1995)

[25] Smirni D Carotenuto M Precenzano F Smirni P Operto FF Marotta R. Memory performances and personality traits in mothers of children with Obstructive Sleep Apnea Syndrome. Psychol Res Behav Manage. (2019) 12:481–7. 10.2147/PRBM.S202469 31303802 PMC6611713

[26] Esposito M Gallai B Parisi L Roccella M Marotta R Lavano SM. Maternal stress and childhood migraine: a new perspective on management. Neuropsychiatr Dis Treat. (2013) 9:351–5. 10.2147/NDT.S42818 23493447 PMC3593768

[27] Operto FF Craig F Peschechera A Mazza R Lecce PA Margari L. Parenting stress and emotional/Behavioral Problems in adolescents with Primary headache. Front Neurol. (2018) 8:749. 10.3389/fneur.2017.00749 29403424 PMC5780397

[28] Achenbach TM Ruffle TM. The child behavior checklist and related forms for assessing behavioral/emotional problems and competencies. Pediatr Rev. (2000) 21:265–71. 10.1542/pir.21-8-265 10922023

[29] Frigerio A Cattaneo C Cataldo M Schiatti A Molteni M Battaglia M Behavioral and emotional problems among Italian children and adolescents aged 4–18 years as reported by parents and teachers. Eur J Psychol Assess. (2004) 20:124–33. 10.1027/1015-5759.20.2.124

[30] Association GA of the WM World Medical Association Declaration of Helsinki: ethical principles for medical research involving human subjects. J Am Coll Dent. (2014) 81:14 10.1515/9783110208856.233 25951678

[31] Kilicoglu AG Mutlu C Bahali MK Adaletli H Gunes H Duman HM. Impact of enuresis nocturna on health-related quality of life in children and their mothers. J Pediatr Urol. (2014) 10:1261–6. 10.1016/j.jpurol.2014.07.005 25164391

[32] Chang SSY Ng CFN Wong SN Group HKCES. Behavioural problems in children and parenting stress associated with primary nocturnal enuresis in Hong Kong. Acta Paediatr. (2002) 91:475–9. 10.1080/080352502317371742 12061366

[33] Sarici H Telli O Ozgur BC Demirbas A Ozgur S Karagoz MA. Prevalence of nocturnal enuresis and its influence on quality of life in school-aged children. J Pediatr Urol. (2016) 12:159–e1. 10.1016/j.jpurol.2015.11.011 26778419

[34] Hassall R Rose J McDonald J. Parenting stress in mothers of children with an intellectual disability: the effects of parental cognitions in relation to child characteristics and family support. J Intellect Disabil Res. (2005) 49:405–18. 10.1111/j.1365-2788.2005.00673.x 15882391

[35] Spratt EG Saylor CF Macias MM Assessing parenting stress in multiple samples of children with special needs (CSN). Fam Syst Heal. (2007) 25:435–49. 10.1037/1091-7527.25.4.435

[36] Tomanik S Harris GE Hawkins J The relationship between behaviours exhibited by children with autism and maternal stress. J Intellect Dev Disabil. (2004) 29:16–26. 10.1080/13668250410001662892

[37] Wiggs L Stores G. Behavioural treatment for sleep problems in children with severe intellectual disabilities and daytime challenging behaviour: effect on mothers and fathers. Br J Health Psychol. (2001) 6:257–69. 10.1348/135910701169197 14596726

[38] Kljajic Z Roje Z Becic K Capkun V. Obstructive sleep apnea in children: how it affects parental psychological status? Int J Pediatr Otorhinolaryngol. (2019) 117:157–62. 10.1016/j.ijporl.2018.11.032 30579072

[39] Smirni D Precenzano F Magliulo RM Romano P Bonifacio A Gison G Inhibition, set-shifting and working memory in Global Developmental Delay preschool children. Life Span Disabil. (2018) 21:191–206.

[40] Smirni D Smirni P Di Martino G Operto FF Carotenuto M. Emotional awareness and cognitive performance in borderline intellectual functioning young adolescents. J Nerv Ment Dis. (2019) 207:365–70. 10.1097/NMD.0000000000000972 30932986

[41] Altiere MJ Von Kluge S Family functioning and coping behaviors in parents of children with autism. J Child Fam Stud. (2009) 18:83 10.1007/s10826-008-9209-y

[42] Dabrowska A Pisula E. Parenting stress and coping styles in mothers and fathers of pre-school children with autism and Down syndrome. J Intellect Disabil Res. (2010) 54:266–80. 10.1111/j.1365-2788.2010.01258.x 20146741

[43] Benson P Karlof KL Siperstein GN. Maternal involvement in the education of young children with autism spectrum disorders. Autism. (2008) 12:47–63. 10.1177/1362361307085269 18178596

[44] Moes D Koegel RL Schreibman L Loos LM. Stress profiles for mothers and fathers of children with autism. Psychol Rep. (1992) 71:1272–4. 10.2466/pr0.1992.71.3f.1272 1480714

[45] Herring S Gray K Taffe J Tonge B Sweeney D Einfeld S. Behaviour and emotional problems in toddlers with pervasive developmental disorders and developmental delay: associations with parental mental health and family functioning. J Intellect Disabil Res. (2006) 50:874–82. 10.1111/j.1365-2788.2006.00904.x 17100948

[46] Carotenuto M Ruberto M Fontana ML Catania A Misuraca E Precenzano F Executive functioning in autism spectrum disorders: a case-control study in preschool children. Curr Pediatric Res. (2019) 23:112–6.

[47] Knussen C Sloper P Stress in families of children with disability: a review of risk and resistance factors. J Ment Heal. (1992) 1:241–56. 10.3109/09638239209005457

[48] Crowe TK Clark L Quails C The impact of child characteristics on mothers' sleep patterns. Occup Ther J Res. (1996) 16:3–22. 10.1177/153944929601600101

[49] Osborne LA McHugh L Saunders J Reed P. Parenting stress reduces the effectiveness of early teaching interventions for autistic spectrum disorders. J Autism Dev Disord. (2008) 38:1092–103. 10.1007/s10803-007-0497-7 18027079

[50] Ferrara P Amodeo ME Sbordone A Ianniello F Verrotti A Petitti T. The impact of motivational therapy in the management of enuretic children. Turkish J Urol. (2018) 44:346–50. 10.5152/tud.2018.50329 29932404 PMC6016659

[51] Peng CC-H Shei-Dei Yang S Austin PF Chang S-J. Systematic review and meta-analysis of alarm versus desmopressin therapy for pediatric monosymptomatic enuresis. Sci Rep. (2018) 8:16755. 10.1038/s41598-018-34935-1 30425276 PMC6233184

